# Global Evidence on the Association between POS Advertising Bans and Youth Smoking Participation

**DOI:** 10.3390/ijerph13030306

**Published:** 2016-03-09

**Authors:** Ce Shang, Jidong Huang, Kai-Wen Cheng, Qing Li, Frank J. Chaloupka

**Affiliations:** 1 Institute for Health Research and Policy, University of Illinois at Chicago, Chicago, IL 60608, USA; jhuang12@uic.edu (J.H.); kcheng@gsu.edu (K.-W.C.); fjc@uic.edu (F.J.C.); 2 Department of Economics, University of Illinois at Chicago, Chicago, IL 60608, USA; 3 Tobacco Center of Regulatory Science, Georgia State University, Atlanta, GA 30303, USA; 4 American Cancer Society, Atlanta, GA 30303, USA; qing.li@cancer.org

**Keywords:** point-of-sale advertising ban, experimental smoking, youth, global youth tobacco survey, MPOWER

## Abstract

*Background*: Point-of-sale (POS) tobacco advertising has been linked to youth smoking susceptibility and experimental smoking. However, there is limited evidence of the association between POS advertising bans and youth smoking participation. This study aims to examine how such bans are associated with current smoking, daily smoking, and regular smoking (≥1 cigarettes per day) participation among youth. *Methods*: one to two waves (primarily one wave) of the Global Youth Tobacco Survey were conducted in 130 countries between 2007 and 2011. These surveys were linked to the WHO “MPOWER” data using country and year identifiers to analyze the association between POS advertising bans (a dichotomous measure of the existence of such bans) and smoking participation in the past month. Weighted logistic regressions were employed to analyze this association while controlling for age, gender, parents’ smoking status, 6 MPOWER policy scores, and GDP per capita. *Results and Conclusions*: We find that in countries with POS advertising bans, current smoking (OR = 0.73, *p* ≤ 0.1), daily smoking (OR = 0.70, *p* ≤ 0.1), and regular smoking (OR = 0.75, *p* ≤ 0.05) participation in the past month is significantly lower, suggesting that POS promotion bans can potentially reduce youth smoking. This study provides evidence to support the implementation of POS promotion regulations by the US FDA and implementation of the WHO FCTC guidelines regarding restrictions on tobacco POS promotion.

## 1. Introduction

As governments impose more stringent restrictions on tobacco advertising in traditional channels such as television, point-of-sale (POS) advertising as a marketing strategy has become more important to the tobacco industry [1,2,3,4,5,6,7]. A number of studies have documented that youth and young adults are more susceptible to tobacco advertising than adults, rendering POS advertising a crucial tool for the industry to attract youth into a potential lifelong addiction and maintain their long-term profits [8,9,10,11,12,13,14,15,16,17,18,19,20,21,22,23,24,25,26,27,28,29,30,31,32,33,34,35,36]. As a matter of fact, POS tobacco promotion, such as advertising and preferred ways of display, is observed to be more prevalent in stores located near schools or in communities with a higher proportion of potential youth consumers than in stores located elsewhere [9,24,29,33,34,36].

The existing evidence further underscores the need for regulating POS advertising by showing increased smoking participation or susceptibility to smoking among youth who were exposed to POS promotion of tobacco products [24,34]. Specifically, a number of studies have shown that youth and young adults who are exposed to POS promotion are 1.1–2.7 times more likely to experiment with smoking [9,18,23,24]. Legislation banning or restricting POS promotion, including advertising and displays, may have significant public health benefits by effectively reducing smoking among young people, and thus future smoking prevalence. Using a simulation model, Levy *et al.* (2015) projected that comprehensive POS restriction may reduce smoking prevalence in the US by approximately 16% by 2065 [37]. 

Despite the importance of regulating POS promotion, such policies are lacking in many countries. According to the World Health Organization (WHO), as of 2012, only 67 out of 225 countries have banned POS advertising, with just around 25% of the world population protected by such bans [38]. There are even fewer countries banning POS displays, and these are primarily developed countries such as Canada and Australia. In the U.S., the 2009 Family Smoking Prevention and Tobacco Control Act (FSPTCA) granted the U.S. Food and Drug Administration (FDA) authority to regulate the manufacture, distribution and marketing of tobacco products, including POS promotion. However, the FDA has yet to propose or implement any POS promotion regulations except for bans on free cigarette samples and selling tobacco products via vending machines [39]. 

Parallel to this policy vacuum at the federal level in the U.S., empirical evidence that examines how regulation of POS promotion perform in tobacco control is very limited. Whereas there are several studies that have investigated the effects of POS promotion bans on smoking outcomes, the findings are inconsistent [9,40,41,42,43]. One study conducted by Scheffels and Lavik in Norway found that following the implementation of a POS promotion ban, a significant proportion of smokers agreed that such bans make it more difficult to choose brands or to buy tobacco and the general public agreed that they make uptake more difficult. However, the proportion of smokers who think such bans help quitting decreased following implementation, suggesting the ban has mixed effects [40]. In addition, one study using Irish data did not find any significant changes in cigarette sales following POS promotion ban, suggesting that the ban was not effective, at least in the short term, in Ireland [41]. In contrast, another recent study employed longitudinal data from the U.S., UK, Australia, and Canada to examine the effects of POS display bans on tobacco use outcomes, and found that these bans lower impulse purchases and non-usual brand purchases due to POS promotion [42]. However, this study did not expand to examine POS advertising bans or other smoking outcomes, and their sample was limited to only adult smokers in these four high-income countries. 

As far as youth smoking is concerned, only two studies sought to examine how youth smoking is associated with POS promotion bans. Using Irish data, McNeil *et al*. (2011) found that there was no significant change in youth smoking prevalence before and after the ban, although it lowers the odds of youth overestimating smoking prevalence among their peers [43]. Shang *et al*. (2015) utilized Global Youth Tobacco Survey (GYTS) data from 130 countries to examine how POS advertising bans are associated with the prevalence of experimental smoking or ever smoking among youth. Results show that POS advertising bans are significantly associated with lower prevalence of experimental smoking among youth [44]. 

Given the very limited and inconsistent empirical evidence with regard to the effectiveness of POS promotion regulation in reducing smoking, especially among youth who are susceptible to advertising, more research is needed in this matter to better inform policy makers about POS promotion regulations and their consequences. This study seeks to expand Shang *et al*. [44] by utilizing GYTS data from 130 countries to examine the association between POS advertising bans and youth smoking outcomes including prevalence of current smoking, daily smoking, and regular smoking. Taking advantage of cross-country comparison using a large number of countries, results from this study will provide important information on the potential effect of POS promotions bans in reducing youth smoking to the U.S. FDA and countries aiming at reducing tobacco use and non-communicable diseases attributable to tobacco use.

## 2. Methods

### 2.1. Data and Measures

#### MPOWER

Following Shang *et al*. (2015), we linked MPOWER policy data with Global Youth Tobacco Survey (GYTS) to carry out the study [44]. Specifically, the information on country-level POS advertising bans during 2007–2012 came from the WHO MPOWER database. The MPOWER measures were introduced by the WHO under the Framework Convention on Tobacco Control (FCTC) guidelines to assist countries in achieving their tobacco control goals using six known tobacco control methods: M (monitor tobacco use), P (protect people from smoke), O (offer help to quit), W (warn about the dangers of tobacco), E (enforce bans on tobacco marketing), and R (raise taxes on tobacco) [45]. The database contains scores that evaluate these six policy methods in 196 countries (a score of 1 represents a lack of data and a score of 2–5 respectively represents the strength of policy from weakest to strongest policy strength) in 2007–2008, 2010, and 2012. In addition to these six policy measures, MPOWER further assesses individual policies on a more detailed scale including POS advertising bans that are part of the E “enforce bans on tobacco marketing” score. During 2007–2012, a majority of the 196 countries did not change their POS advertising policies. For those that did, we cross-examined the Euromonitor and ERC tobacco reports on tobacco control policies to obtain the timing of the policy change. Finally, we constructed time series data of POS advertising bans for each year during 2007–2012, measured by a dichotomous variable which equals 1 if the country has such a ban, and 0 otherwise. For the six MPOWER scores, we made the assumption that these scores in 2009 and 2011 did not change from the ones in the previous year and created an annual time series of data of these scores between 2007 and 2012, which were used as controls for the tobacco control environment in the analyses. 

#### 2.2. Global Youth Tobacco Survey (GYTS)

Individual level data came from the GYTS, which are school-based surveys designed to monitor tobacco use among youth on a global basis and to guide the implementation and evaluation of tobacco control policies. While GYTS were sporadically carried out in many countries between 2003 and 2011, most countries were only surveyed once during 2007–2011 when MPOWER data were available. In fact, among the 130 countries that were identified with the MPOWER policy information and included in the analyses, a majority of countries (*N* = 107) were surveyed only once, with 17 countries surveyed two to three times and the remaining 6 countries surveyed more than once but in different subnational regions.

GYTS contains questions about smoking behavior by students, including “in the past month, on how many days did you smoke cigarettes?” and “in the past month, on days you smoked, how many cigarettes did you smoke?”. Using these two questions, we constructed three dichotomous smoking outcomes, including current smoking participation (smoked in past month), daily smoking participation (smoked daily in past month), and regular smoking participation (≥1 cigarette per day on days they smoked). We could not construct cigarette consumption because responses to smoking-related questions were categorical and recorded intervals rather than numbers. Nonetheless, the constructs measure both frequency and intensity of youth smoking. 

GYTS data also provides a list of demographic variables that were controlled for in the analyses. These include respondents’ age, gender, and their parents’ smoking status. In addition, we controlled for the real GDP per capita of each country obtained from the World Development Indicators (WDI) data. Table 1 presents the definitions of individual-level smoking status and demographic variables. The definitions of country-level variables such as the dichotomous measure of POS advertising bans, 6 MPOWER policy scores, and GDP per capita can be found in one previous study by Shang *et al*. (2015) [44].

### 2.3. Methodology

We used similar methods to those employed in Shang *et al*. (2015) to study the association between POS advertising bans and youth smoking participation [44]. POS advertising bans and six MPOWER scores were linked to GYTS data between 2007 and 2011 using year and country identifiers. Logistic regressions were employed to analyze the association between smoking outcomes (current smoking, daily smoking, and regular smoking) and POS advertising bans, while controlling for age, gender, parents’ smoking status, six MPOWER scores, and GDP per capita. We estimated the variance inflation factor (VIF) to evaluate whether the country-level controls (six MPOWER scores and GDP per capita) are highly correlated with POS advertising bans. The estimates show that the magnitude of VIF in the current specification is only 1.6, far lower than 5 and 10-the rule-of-thumb values for high multicollinearity—suggesting that these controls do not incur the multicollinearity problem and can be included in the model. Because MPOWER scores measure both lack of data and policy strength levels, we included additional controls indicating the lack of data to disentangle the effects of missing records or data from the effects of policies. As aforementioned, most countries were only surveyed once during 2007–2011. Therefore, analyses were based on a comparison of smoking outcomes between countries with and without POS bans using cross-sectional data and, as a result, country and year fixed effects were not controlled for. Nevertheless, we adjusted for country-specific attributes using MPOWER scores and GDP per capita, and estimated standard errors clustered at the country level to account for the correlations between individuals from the same country. Following previous cross-country studies, analytical weights were constructed using GYTS final weights rescaled to the sample size of each country to account for the sample composition and survey methodologies [42,46,47,48]. All analyses were conducted using Stata v. 13.0.1. (Stata Corp LP, College Station, TX, USA). 

## 3. Results

**Figure 1:**
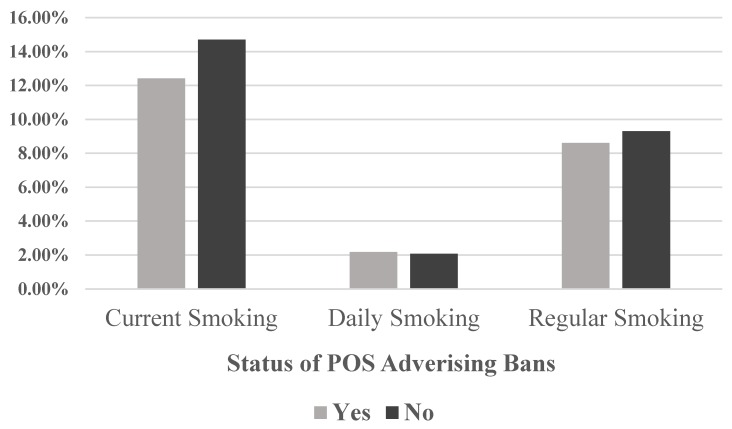
Current smoking, daily smoking, and regular smoking by POS advertising bans.

In Figure 1, we illustrate the unadjusted rates of current smoking, daily smoking, and regular smoking participation among youth, using the status of POS advertising bans. Current smoking and regular smoking are lower in countries with POS advertising bans than in countries without such bans, while daily smoking participation is about the same. On average, current smoking and regular smoking (consuming ≥1 cigarettes when youth smoke) are more prevalent than daily smoking in the past month, suggesting that many youth are intermittent smokers and not yet conformed daily smokers. 

In Table 2, we present the weighted summary statistics for dependent and independent variables. The prevalence of current smoking, daily smoking, and regular smoking among youth is 14%, 2%, and 9%, respectively. The age distribution shows that each age category accounts for about 10%–20% of the sample. Parents’ smoking status suggests that, while about 60% of respondents reported that neither parents smoke, 7% reported that both parents smoke, 25% reported that their father smokes, and 4% reported that their mother smokes. Among the youth respondents, 24% live in a country with POS advertising bans. The means of MPOWER scores are mostly between 2 and 3, suggesting that some countries implement none while some countries implement policies with a higher strength. The average per capita income in these primarily low- and middle-income countries is $4709. 

Table 3 contains the results for the association between POS advertising bans and smoking outcomes, estimated using odds ratios. POS advertising bans are significantly associated with lower odds of smoking (OR = 0.73, *p* ≤ 0.1), daily smoking (OR = 0.70, *p* ≤ 0.1), and regular smoking (OR = 0.75, *p* ≤ 0.05) in the past month. Compared with youth aged 11 or younger, those aged 15 years or older are more likely to have smoked in the past month. In addition, youth who have at least one smoking parent are more likely to smoke, compared with those whose parents do not smoke. 

We further conducted several robustness checks to examine the validity of these estimates. First, because the six composite scores in the years 2009 and 2011 are not available from the MPOWER data, we used prior years’ policy scores for these two years (as in the analyses presented in Table 2). Therefore, to examine whether our results are sensitive to this approach, we also conducted analyses by using the scores from the subsequent years (2010 and 2012), and by randomly assigning the scores from either the prior or the subsequent year. The marginal effect and elasticity estimates from the above three approaches are presented in Table 4, with results in the first three columns showing estimates from our primary models and results in the rest columns showing estimates from the sensitivity analyses. 

As reported in the table, these results show that POS advertising bans are associated with a four percentage-point or 27% lower current smoking prevalence, a one percentage-point or 35% lower daily smoking prevalence, and a two percentage-point or 26% lower regular smoking prevalence among youth. These findings are robust to the variety of ways of controlling for the tobacco control environment using MPOWER scores. We conducted additional sensitivity analyses by not applying analytical weights in the regressions. The results remain very similar.

## 4. Discussions 

In summary, this study assesses the association between POS advertising bans and smoking outcomes among youth and provides evidence that such bans are associated with lower prevalence of current smoking, daily smoking, and regular smoking. Specifically, these findings suggest that in countries with POS advertising bans, the prevalence of current smoking, daily smoking, and regular smoking is 27%, 26%, and 35% lower, respectively, which is consistent with previous findings that POS advertising bans are associated with a 31% lower probability of experimental smoking [44], and that exposure to POS promotion is associated with 1.6 times higher odds of experimental smoking and 1.3 times higher odds of smoking susceptibility among youth [8]. These results also illustrate that the potential effect of POS advertising bans is greater with respect to reducing experimental or regular smoking (≥1 cigarettes per day) than in reducing smoking participation and daily smoking, which corroborate with the broader literature that indicates that POS promotion induces impulse purchase and use of cigarettes [49,50,51,52]. In other words, whereas the implementation of POS promotion bans may reduce smoking in many stages, it is more likely to reduce smoking through reducing impulse purchase and thus through reducing experimental or occasional smoking which may progress into established or more intensive and frequent smoking. 

Furthermore, because our sample primarily consists of low- and middle-income countries, findings from this study suggest that POS advertising bans are associated with lower youth smoking participation in these countries. This evidence supports the comprehensive tobacco control measures proposed by the WHO FCTC that include POS promotion bans. In addition, whereas several studies from high-income countries show that POS advertising is significantly associated with higher youth smoking participation, one study by Kim *et al.* suggested that it is not significantly associated with youth frequent smoking or cigarettes per day [12,24,30]. We instead find that POS advertising bans are significantly associated with lower daily smoking and regular smoking (≥1 cigarettes per day), indicating that the effect of POS advertising bans in reducing youth smoking is likely to be more pronounced in low- and middle-income countries. 

In these analyses, the association between POS advertising bans and youth smoking outcomes was estimated by comparing these outcomes between countries with and without such bans. As a result, these estimates do not identify the causal effects of POS advertising bans in reducing smoking and are likely to be the upper bound estimate because bans imposed as part of a broader or more comprehensive tobacco control package could interact with other policies and potentially inflate the estimated effects of POS advertising bans. Nevertheless, these estimates are likely to reflect the cumulative effects of POS advertising bans over a period of time and support the projection that comprehensive POS restriction may reduce smoking prevalence in the U.S. by approximately 16% by 2065 [37].

Like many other studies, there are some limitations in this study. As aforementioned, we do not have multiple waves of cross-sectional data to examine how changes in POS advertising bans impact smoking behavior. Therefore, results in this paper only illustrate the association between POS advertising bans and reduced youth smoking participation, rather than the causal effect of such bans in reducing youth smoking. Second, because GYTS were conducted in a large number of countries, some demographic characteristics such as family income, allowance, and peer influence were not controlled for due to lack of data or inconsistent measures. Smoking outcomes in the past month were self-reported and may contain measurement errors. However, despite these limitations, our results are robust to various methods of constructing tobacco control environment controls, whether regressions were weighted, and whether the second wave for a limited number of countries was included in the sample. Our findings shed light on the effects of POS promotion bans in reducing smoking and offer upper bound estimates of such effects. Future research is needed to identify the casual effects of POS promotion bans on smoking and quantify their impact. 

## 5. Conclusions

In conclusion, the findings of this study provide evidence that POS advertising bans are significantly associated with lower current smoking, daily smoking, and regular smoking participation among youth. These findings are consistent with the existing literature showing that POS promotion increases youth smoking participation in the US and in many other countries. However, to date, POS promotion regulation has not yet been implemented at the federal level in the U.S. As the FDA has the authority to regulate the marketing of tobacco products, they may consider POS promotion regulations to curb tobacco use among youth. Moreover, this study also indicates that POS promotion regulations are potentially more effective in reducing youth smoking in low- and middle-income countries than in high-income countries, supporting the implementation of WHO FCTC guidelines regarding restrictions on tobacco POS promotion in many countries.

## Figures and Tables

**Table 1:** Variable definition: Individual-level smoking outcomes and demographic characteristics.

Variable Name	Description
*Individual-level variables*	
Current smoking	Binary variable for smoking cigarettes in the past month.
Daily smoking	Binary variable for smoking cigarettes daily in the past month.
Regular smoking	Binary variable for smoking ≥1 cigarettes on days when they smoked in the past month.
Age dummies	Binary indicators for 8 age categories (≤11 years as old-referent) from age 12 to 16, and ≥17
Parents’ smoking status	Binary indicator for 5 categories of parents’ smoking status (neither as referent), both, father only, mother only and missing/don’t know

Note: Please see Shang *et al.* (2015) for definitions of country-level variables including POS advertising ban dummy, MPOWER scores, and GDP per capita [44].

**Table 2:** Weighted summary statistics (*N* = 569,853).

Individual-Level	Mean	(95% C.I.)	Country-level	Mean	(95% C.I.)
Current Smoking	0.142	(0.122, 0.161)	POS ad ban	0.240	(0.145, 0.335)
Daily Smoking	0.021	(0.016, 0.026)	M score	2.825	(2.647, 3.002)
Regular Smoking	0.091	(0.078, 0.105)	M no data	0.091	(0.039, 0.143)
Age dummies			P score	2.527	(2.317, 2.737)
≤11	0.061	(0.037, 0.084)	P no data	0.068	(0.000,0.149)
12	0.089	(0.074, 0.105)	O score	3.305	(3.171, 3.438)
13	0.187	(0.163, 0.210)	W score	2.674	(2.424, 2.923)
14	0.226	(0.206, 0.246)	E score	3.135	(2.898, 3.372)
15	0.204	(0.185, 0.222)	R score	3.380	(3.193, 3.566)
16	0.121	(0.104, 0.138)	R no data	0.017	(0.000,0.038)
≥17	0.070	(0.047, 0.093)	GDP per capita	4.709	(3.621, 5.797)
Missing	0.043	(0.000,0.102)	--	--	--
Parent smoking status			--	--	--
Both	0.070	(0.056, 0.083)	--	--	--
Father only	0.252	(0.219, 0.285)	--	--	--
Mother only	0.040	(0.030, 0.050)	--	--	--
Neither	0.566	(0.531, 0.601)	--	--	--
Don’t know	0.072	(0.041, 0.104)	--	--	--
Male	0.494	(0.487, 0.501)	--	--	--

**Table 3:** Weighted logistic regressions, the association between POS ad bans and current smoking, daily smoking, and regular smoking participation.

Variables	Current Smoking		Daily Smoking		Regular Smoking	
	AOR	(95% C.I.)	AOR	(95% C.I.)	AOR	(95% C.I.)
**POS ad ban**	0.727 *	(0.528, 1.003)	0.699 *	(0.482, 1.014)	0.749 **	(0.562, 0.999)
Age Dummies						
12	1.160	(0.559, 2.409)	1.420	(0.320, 6.294)	1.178	(0.456, 3.043)
13	0.852	(0.519, 1.398)	0.591	(0.265, 1.318)	0.845	(0.474, 1.505)
14	1.104	(0.693, 1.760)	0.940	(0.462, 1.913)	1.188	(0.694, 2.034)
15	1.533 *	(0.969, 2.426)	1.785 *	(0.929, 3.430)	1.759 **	(1.039, 2.977)
16	2.074 ***	(1.318, 3.263)	3.104 ***	(1.726, 5.580)	2.471 ***	(1.487, 4.108)
17	2.756 ***	(1.760, 4.316)	5.121 ***	(2.761, 9.498)	3.391 ***	(2.042, 5.632)
Missing	1.005	(0.424, 2.381)	1.341	(0.664, 2.707)	1.231	(0.557, 2.719)
Parents’ Smoking						
Both	3.346 ***	(2.882, 3.884)	4.911 ***	(4.061, 5.939)	3.497 ***	(3.025, 4.043)
Father only	1.762 ***	(1.600, 1.940)	1.969 ***	(1.746, 2.222)	1.857 ***	(1.690, 2.039)
Mother only	2.857 ***	(2.438, 3.347)	3.814 ***	(3.182, 4.571)	3.117 ***	(2.675, 3.631)
Don’t know	1.839 ***	(1.405, 2.409)	2.545 ***	(1.742, 3.719)	2.054 ***	(1.532, 2.754)
Other Controls						
Male	1.683 ***	(1.406, 2.015)	1.702 ***	(1.408, 2.058)	1.684 ***	(1.410, 2.011)
M score	0.957	(0.806, 1.137)	1.011	(0.799, 1.278)	0.961	(0.814, 1.134)
M score missing	0.709 *	(0.475, 1.058)	0.555 *	(0.301, 1.025)	0.605 **	(0.403, 0.909)
P score	1.058	(0.923, 1.213)	1.028	(0.850, 1.243)	1.055	(0.911, 1.221)
P score missing	0.985	(0.555, 1.750)	1.290	(0.539, 3.086)	1.072	(0.566, 2.028)
O score	0.992	(0.762, 1.291)	1.297 **	(1.010, 1.665)	1.137	(0.932, 1.386)
W score	1.066	(0.855, 1.328)	0.931	(0.778, 1.113)	0.979	(0.851, 1.126)
E score	1.019	(0.885, 1.173)	1.343	(1.111, 1.624)	1.093	(0.954, 1.251)
R score	1.450 ***	(1.269, 1.657)	1.528 ***	(1.252, 1.865)	1.473 ***	(1.284, 1.690)
R score missing	4.366 ***	(1.917, 9.943)	7.525 ***	(2.321,24.400)	4.911 ***	(1.833,13.157)
GDP per Capita	1.022 ***	(1.007, 1.037)	1.037 ***	(1.019, 1.055)	1.028 ***	(1.013, 1.043)

Note: * <0.1, ** <0.05, *** <0.01 in two-tailed test.

**Table 4:** Marginal effects and elasticity, the Association between POS ad bans and current smoking, daily smoking, and regular smoking participation.

Variables	Current Smoking			Daily Smoking			Regular Smoking		
	Prior	Later	Random	Prior	Later	Random	Prior	Later	Random
Marginal Effect	−0.036 *	−0.040 *	−0.038 *	−0.007 *	−0.007 *	−0.007 *	−0.022 **	−0.024 **	−0.023 **
(S.E.)	(0.019)	(0.020)	(0.019)	(0.004)	(0.004)	(0.004)	(0.011)	(0.011)	(0.011)
Elasticity	−0.273 *	−0.307 *	−0.286 *	−0.350 *	−0.340 *	−0.341 *	−0.262 **	−0.279 **	−0.266 **
(S.E.)	(0.139)	(0.151)	(0.143)	(0.184)	(0.186)	(0.182)	(0.132)	(0.131)	(0.129)

Note: * <0.1, ** <0.05 in two-tailed test. Columns with headers “prior”, “Later”, and “random” show results from models using different methods to fill in missing MPOWER scores.
